# Management of diabetic eye disease: an overview

**Published:** 2015

**Authors:** Nicholas Beare

**Affiliations:** Consultant Ophthalmologist: St Paul's Eye Unit, Royal Liverpool Hospital. Liverpool, UK.

**Figure:**
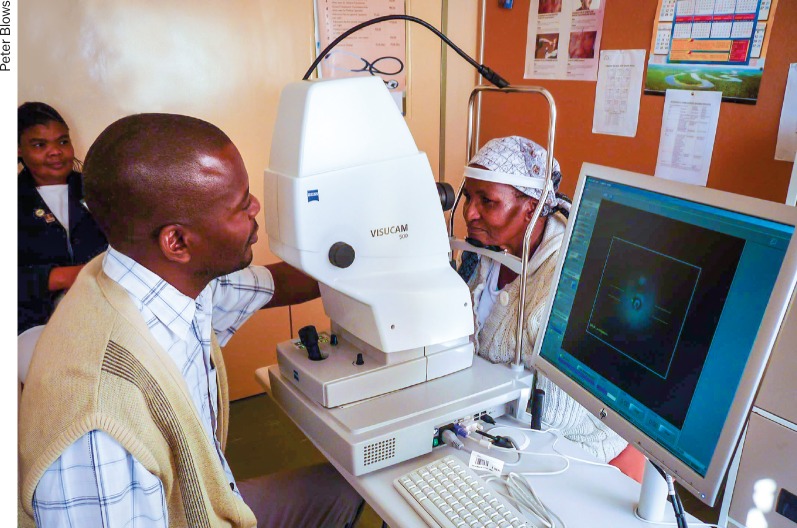
Photographie screening for diabetic eye disease can identify patients who will benefit from laser treatment. BOTSWANA

**Figure:**
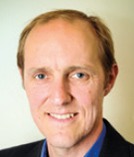
Nicholas Beare

## Systemic risk factors

In order to reduce the risk of diabetic eye disease (both retinopathy and maculopathy) progressing and causing visual loss, it is important for all people with diabetes to maintain good overall health and good control over their diabetes. This is especially important for patients who already have diabetic retinopathy (DR) which is already affecting their vision, or is likely to damage it soon.

The two most important risk factors are high blood glucose (sugar) and high blood pressure.

High cholesterol and lipids also seem to be related to DR getting worse. Treatment of high cholesterol and lipids with statin medications, if available, reduces the risk of DR progressing. Maintenance of a healthy lifestyle overall will be beneficial for DR. This includes not smoking (or giving up) and getting regular exercise. People with diabetes should follow a healthy diet and avoid sugar and refined carbohydrates as much as possible.

Eye health professionals have a role in identifying patients at risk of sight loss from DR, and reinforcing messages about diabetes control and healthy living. Screening for DR and laser treatment for DR are both good opportunities for eye health professionals to get these messages across to patients.

## Laser for DR and maculopathy

Laser is the mainstay of treatment for both DR and maculopathy. Table 1 summarises the indications, desired response indicators, insufficient response indicators and side effects of both, based on the articles on preventing sight loss from DR and preventing sight loss from maculopathy, on pages 65 and 67 respectively. There is a glossary of terms on page 65 and the poster in the centre of this issue (pages 70–71) provides helpful background about the terms used.

**Tips for successful laser**

- Make sure that the patient has realistic expectations and is aware of the limitations.
- Ensure the laser focus and optical focus are together.
- Take care focusing the laser (aiming beam) and titrating the power.
- For pan-retinal laser, ensure that the temporal quadrant is adequately treated, up to the edge of the macula.
- For macular laser:
  – cover the area of diabetic macular oedema (DMO) systematically, as laser uptake may be poor in the area of DMO
  – if the fovea is indistinct, laser outside the area which contains the fovea within it.
- If attaining appropriate burn strength becomes difficult or variable, remove the contact lens and reapply coupling gel.

**Table 1.** Indications, response indicators and side effects of laser treatment for maculopathy and retinopathy

	Laser for diabetic retinopathy (DR): peripheral retinal photocoagulation (PRP)	Laser for maculopathy (focal or grid laser)
**Indications**	Severe pre-proliferative DR 4-2-1 rule (see page 65) Proliferative DR Proliferative DR with high-risk characteristics (new vessels or vitreous haemorrhages)	Clinical significant macular oedema (CSMO) Diabetic macular oedema (DMO) affecting the central fovea Exudates threatening/affecting vision
**Desired response**	Regression of new vessels Prevention of new vessel formation	Reduction in DMO Prevention of (further) deterioration of vision
**Insufficient response?**	Reapply PRR, making burns more dense and extensive Keep repeating	Once grid is complete over oedematous area (or macula in diffuse DMO) no benefit from further grid laser. Individual microaneurysms can be targeted.
**Side effects and complications**	Reduction in night vision and peripheral vision Initiating/worsening DMO Foveal burn (rare)	Foveal burn Paracentral scotomas for deliberately close laser shots

## Glossary

### Clinically significant macular oedema (CSMO)

CSMO is when leakage from small retinal blood vessels causes macular oedema (retinal swelling) and exudates (fat deposits from the blood) which are sufficiently close to the fovea (central macula) to affect or threaten the vision.

### Diabetic maculopathy

Diabetic maculopathy is part of diabetic retinopathy. Maculopathy is damage to the macula, the part of the eye responsible for central vision.

### Diabetic macular oedema (DMO)

Diabetic macular oedema occurs when blood vessels near to the macula leak fluid or protein onto the macula.

### Diabetic retinopathy (DR)

Diabetic retinopathy occurs when changes in blood glucose levels cause changes in retinal blood vessels. In some cases the vessels leak fluid into the macula part of the retina which swells up (DMO). In other cases, abnormal blood vessels will grow on the surface of the retina.

### Peripheral retinal photocoagulation (PRP)

Cauterisation of the peripheral retina using laser, with a minimum of 2,000 effective burns.

### Proliferative diabetic retinopathy

This is the advanced stage of diabetic retinopathy. New blood vessels grow along the inside surface of the retina and into the vitreous gel, the fluid that fills the eye. These vessels are fragile and more likely to leak and bleed. Scar tissue is formed and can contract and cause retinal detachment (the pulling away of the retina from underlying tissue) – which results in blindness.

## The Early Treatment in Diabetic Retinopathy Study (ETDRS)

The Early Treatment in Diabetic Retinopathy study (ETDRS) has produced over 20 publications. They are listed at **https://clinicaltrials.gov/ct2/show/study/NCT00000151**

ETDRS and the preceding Diabetic Retinopathy Study are summarised in Chapters 1 and 2 of *Clinical Trials in Ophthalmology*, Eds PJ Kertes and MD Conway. Lippincott Williams and Wilkins 1998.

